# Effects of nutraceutical intervention on serum proteins in aged rats

**DOI:** 10.1007/s11357-020-00174-4

**Published:** 2020-03-10

**Authors:** Samantha M. Portis, Dale Chaput, Beau Burroughs, Charles Hudson, Paul R. Sanberg, Paula C. Bickford

**Affiliations:** 1 Department of Neurosurgery and Brain Repair, Center of Excellence for Aging and Brain Repair, USF Morsani College of Medicine, University of South Florida, Tampa, FL 33612 USA; 2 Proteomics and Mass Spectrometry Facility, College of Arts and Sciences, University of South Florida, Tampa, FL 33612 USA; 3 Proteomics Core Facility, College of Medicine, University of South Florida, Tampa, FL 33612 USA; 4 James A. Haley VA Hospital, Research Service, 13000 Bruce B Downs Blvd, Tampa, FL 33612 USA

**Keywords:** Aging, Inflammation, Bioinformatics, Proteomics, Serum

## Abstract

Aging is associated with many pathophysiological changes that could lead to the onset of degenerative disease. Some of the physiological changes that occur with aging include increased inflammation and decreased stem cell proliferation, leading to decreased capacity for tissue regeneration and loss of function. In previous studies, we and others have found nutraceutical intervention to ameliorate some of the deleterious effects associated with aging. In particular, we have previously shown that NT-020, a supplement composed of a proprietary blend of blueberries, green tea, vitamin D3, and carnosine, is able to rescue age-related cognitive deficits, impaired neurogenesis, and inflammation in rats. We have also previously demonstrated that stem cells cultured with old serum showed decreased proliferation; however, when stem cells were cultured in serum from old rats given a diet supplemented with NT-020, proliferation did not differ from that of cells cultured with serum from young rats. While it is clear that NT-020 is exerting a therapeutic, anti-aging effect, the mechanisms of action were yet to be fully elucidated.To that end, in the present study, we conducted a bioinformatics experiment to examine the rat proteome of serum from young and old control rats and young and old rats given a diet supplemented with NT-020. Serum from old rats showed an increase in some inflammatory and pro-aging factors while serum from old rats given a diet supplemented with NT-020 showed an increase in some anti-aging factors, most notably proteins associated with the complement system and autophagy. A number of immune functions that increase with age were shown to be downregulated with NT-020 treatment.

**Electronic supplementary material:**

The online version of this article (10.1007/s11357-020-00174-4) contains supplementary material, which is available to authorized users.

## Introduction

As the population ages, there is increased susceptibility to the onset of disease and age-related degenerative conditions, making aging a central area of interest in medical sciences research (Wagster et al. 2012; Flowers et al. 2016). Aging is a complex and multifactorial physiological process associated with decreased stem cell proliferation in stem cell niches throughout the body, leading to reduced tissue rejuvenation and loss of organ function (Murshid et al. 2013; Bickford et al. 2015). The decline in stem cell regeneration is global, occurring both in the periphery (Ning et al. 2019; Jung et al. 2019; Chambers et al. 2007) and in the central nervous system (CNS) (Villeda et al. 2011; Lazarov et al. 2010; Kozareva et al. 2019; Ayaz et al. 2019; Kase et al. 2019; Stankiewicz et al. 2019; Bickford et al. 2015; Flowers et al. 2015; Acosta et al. 2010). While the exact biological mechanisms underlying dampened stem cell proliferation with age remain unclear, two areas of investigation are being explored: cell-autonomous changes and cell non-autonomous changes. Cell-autonomous changes involve the senescence of genes such as p53, p16^INK4A^, critical proteins involved in the Wnt/β-catenin pathways, and others (Sharpless and DePinho 2007; Bickford et al. 2015). Cell non-autonomous effects on stem cell niches refer to various factors, such as pro-inflammatory cytokines, chemokines, and other proteins that may influence the microenvironment (Bickford et al. 2015). Very salient early examples of circulating factors impacting stem cell proliferation come from heterochronic parabiosis studies. Heterochronic parabiosis entails the surgical union of the circulatory system of a young mouse with an old mouse. It was observed that exposure to blood from old mice had a deleterious impact on stem cell proliferation in young mice. Conversely, exposure to blood from young mice had a positive effect on stem cell proliferation in old mice, thus demonstrating how the systemic milieu can impact stem cell proliferation (Conboy et al. 2005; Villeda et al. 2011). Additionally, Villeda et al. (2011) injected plasma from either old or young mice into young mice four times over 10 days and subjected the animals to contextual fear conditioning and the radial arm water maze. Young animals injected with plasma from old mice displayed significantly less freezing behavior in the contextual fear conditioning paradigm and made a greater number of errors in the radial arm water maze paradigm, suggesting impaired learning and memory following intravenous injection with old plasma. These studies further highlight how the aged systemic milieu can impact the CNS by exerting a dampening effect on hippocampal neurogenesis. In addition to these in vivo studies, the negative impact of the aged systemic milieu on stem cell proliferation has been reproduced in vitro with various types of stem cells derived from mice or rats being cultured with serum from old animals (Bickford et al. 2015; Villeda et al. 2014).

Many different factors from old blood have been identified as proteins of interest in terms of their potential to impact stem cell proliferation. In their initial study, Villeda et al. (2011) proposed that increased CCL11 (eotaxin) in old blood may be a key regulator impinging upon the cellular microenvironment. Another negative regulator that was proposed is beta2-microglobulin, a pro-aging factor (Smith et al. 2015). Additionally, at the same time, loss of protective factors has also been implicated in aging. Several studies have found that there is decreased expression of growth differentiation factor 11 (GDF-11) with age (Katsimpardi et al. 2014; Sinha et al. 2019). Decreased expression of nuclear factor erythroid 2-related factor 2 (Nrf2) has also been observed with aging. Nrf2 is a transcription factor that regulates the expression of antioxidant proteins and, if it is downregulated with age, this is consistent with the known age-related increase in oxidative stress (Huang et al. 2019; Fulop et al. 2018; Flowers et al. 2016).

Polyphenols have been shown to rescue age-related dysfunctions within the CNS including chronic inflammation, impaired synaptic plasticity, and decreased neurogenesis (Zhu et al. 2008; Ayaz et al. 2019; Flowers et al. 2015; Dias et al. 2012; Rahman et al. 2006; Vauzour 2012). Furthermore, polyphenolic compounds have also been shown to have beneficial effects in mouse models of Alzheimer’s disease (AD) (Rezai-Zadeh et al. 2005; Rezai-Zadeh et al. 2009; Rezai-Zadeh et al. 2008; Singh et al. 2008). Our group has demonstrated that a proprietary blend of polyphenolic compounds including epigallocatechin gallate (EGCG) from green tea, blueberry extract, vitamin D3, and carnosine, called NT-020, attenuates inflammation, enhances hippocampal neurogenesis, and improves cognitive function in old rats (Flowers et al. 2015; Acosta et al. 2010). In a rat model of stroke, rats given an NT-020-supplemented diet showed increased neurogenesis compared with vehicle-treated rats (Yasuhara et al. 2008). In in vitro studies, NT-020 and the blue-green algae spirulina were shown to synergistically increase proliferation of human adult stem cells (Bickford et al. 2006; Bachstetter et al. 2010; Shytle et al. 2010). Furthermore, when rat mesenchymal stem cells (MSCs) or neural progenitor cells (NPCs) were cultured in serum derived from old rats, decreased proliferation was observed. However, if serum from old rats given a diet of chow supplemented with NT-020 was added to the culture, these effects were reversed (Bickford et al. 2015). Older adults given NT-020 supplementation in pill form in a double-blind placebo-controlled clinical trial demonstrated improved cognitive function on a battery of cognitive tests compared with participants in the placebo arm of the study, demonstrating the translational nature of nutraceutical research and effectiveness of NT-020 supplementation (Small et al. 2014). While some of the mechanisms of action for the therapeutic effects of NT-020 have been defined, such as the downregulation of pro-inflammatory cytokines and upregulation of anti-inflammatory cytokines, a complete profile of circulating factors has not yet been generated.

The goal of the current study was to generate a complete list of factors from the systemic milieu that could be altered as a consequence of aging and rescued by NT-020 supplementation. We used bottom-up, discovery-based mass spectrometry proteomics, and a bioinformatics program called Ingenuity Pathway Analysis (IPA, Qiagen) to create a profile for the entire proteome for serum derived from old rats given NT-020 or a normal diet and young rats given NT-020 or a normal diet. Our data suggest that there are age-related molecular changes that can be rescued by NT-020 supplementation.

## Methods

### Animals

Male Fisher rats 3–6 months (young) or 20–22 months (old) of age were obtained from the National Institute on Aging (NIA) contract colonies and were housed at the University of South Florida (USF) AAALAC-accredited animal facility at the Morsani College of Medicine. All experimental procedures were approved by the Institutional Animal Care and Use Committee (IACUC). The rats were fed either a standard NIH-31 chow or a NIH-31 chow supplemented with NT-020 (Natura Therapeutics, Inc.) ad libitum at 135 mg/kg/day for 28 days. Rats were divided into three experimental groups (*n* = 10 per group): old rats given standard NIH-31 chow (old control), old rats given NT-020 supplementation (old diet), and young rats given standard NIH-31 chow (young). Body weight and food consumption were monitored throughout the week and measured three times per week. No differences were observed in either body weight or food consumption for any of the groups. Rats were sacrificed by CO_2_ euthanasia after 28 days. Blood was collected by cardiac puncture into serum collection tubes. Tubes were spun at 3000 rpm for 10 min, and serum was then aliquoted and frozen at − 80 °C until use.

### Sample preparation

In order to prepare the serum samples for analysis on the mass spectrometer, the most abundant proteins had to be depleted from the samples. A volume of 10 μL of sample was added to each spin column containing resin filters from a High Select Top 14 Abundant Protein Depletion Kit (Thermo Fisher Scientific, Inc., P/N A36369). Sample was pipetted over the resin spin columns from the kit, washed three times with wash buffer, and combined. Protein quantitation assays were carried out, and samples were diluted 1:5–1:20 in H_2_O. Bovine serum albumin (BSA) was used as a standard, and 10 μL of sample were added to each well of a 96-well plate. Pierce 660 Assay Reagent (150 μL) supplemented with ionic detergent compatibility reagent (IDCR) was added to the plate. Absorbance was read at 660 nm using a plate reader.

Serum protein samples were buffer exchanged and digested by filter aided sample preparation (FASP). Samples were loaded onto FASP filters with 200 μL of urea buffer and centrifuged at 14,000×*g* for 15 min. An additional 200 μL of urea buffer was added to the sample reservoir and centrifuged again for 15 min. Proteins were then alkylated with 100 μL of 100 mM iodoacetamide (IAA) in the dark for 20 min, followed by centrifuging at 14,0000×*g* for 15 min. After alkylation, samples were buffer exchanged with three additions of urea, followed by three additions of ammonium bicarbonate (ABC); samples were centrifuged at 14,000×*g* for 15 min after each addition. Finally, proteins were digested by adding Trypsin/Lys-C in a 1:50 ratio of trypsin to protein (w:w) and incubated overnight at 37 °C. After incubation, peptides were collected by adding 40 μL of ABC buffer and centrifuging at 14,000×*g* for 10 min. This was repeated for a total of two additions of ABC, before adding 50 μL of NaCl and again centrifuging for 15 min. Lastly, 5 μL of formic acid was added to acidify the samples before desalting.

Following tryptic digest, peptide samples were desalted using C18 SPE columns placed on a vacuum manifold. The C18 SPE columns were activated with 1 ml of acetonitrile (ACN), and then equilibrated with two volumes of H_2_O/0.1% formic acid (FA Sol). Once equilibrated, sample was loaded onto the C18 SPE columns, and then washed/desalted with three volumes of FA Sol. The flow-through collection tubes were then replaced with microcentrifuge tubes for peptide collection. Peptides were eluted with two additions of 500 μL of elution buffer (90:10 ACN:H_2_O + 0.1% formic acid). Samples were dried completely in a vacuum concentrator and then resuspended in 1%ACN/99%H_2_O/0.1% formic acid.

### LC-MS/MS

Peptides were separated on a 50-cm reversed-phase C18 UHPLC analytical column using an EASY-nLC 1200 HPLC (Thermo Fisher Scientific) and analyzed on a hybrid-quadrupole-Orbitrap mass spectrometer (Q Exactive HF-X, Thermo Fisher Scientific) with a 120 min gradient. Full MS survey scans were acquired at 60,000 resolution. Each sample was analyzed twice, once using a “traditional data-dependent acquisition (DDA)” method, and once using a “segmented DDA” method. The “traditional DDA” method selected the top 30 most abundant peptides across the full mass scan range of 400–1600 m/z. The “segmented DDA” method selected the most abundant peptides across 3 different mass scan ranges 375–600 m/z (top 20), 600–800 m/z (top 10), and 800–1200 m/z (top 10). Raw data files for each method were searched as technical replicates and combined.

### Analysis

Raw data files were searched using the MaxQuant software (Max Planck Institute of Biochemistry) against the current *Rattus norvegicus* proteome database from Uniprot. Parameters included the fixed modification of cysteine by carbamidomethylation, as well as the variable modifications methionine oxidation and protein N-terminal acetylation. A 1% false discovery rate was used for both peptide and protein identification. The MaxQaunt LFQ feature was used for label free normalization.

The Perseus software (Max Planck Institute of Biochemistry) was used for the filtering and imputation of proteomics data as described previously (Tyanova et al. 2016). Briefly, label free quantification intensities (LFQ) were used as expression values and log transformed. The data were filtered to include proteins that were identified with LFQ intensities (valid values) in at least 60% of samples within at least one treatment group, and missing values were then imputed using the default settings. Samples that showed a skewed, non-normal distribution following imputation were excluded from further analysis. The following number of samples remained per treatment group: young control (*n* = 6), old control (*n* = 7), and old diet (*n* = 5).

Following imputation in Perseus, the data were exported, and Excel was used to generate ratios between comparisons, as well as calculate Welch’s *t* test *p* values, and *z* scores, as previously described (Flowers et al., 2017). Briefly, ratios were generated using the LFQ intensity values for each comparisons of interest. Proteins with a Welch’s *t* test *p* < 0.05, determined using log2-transformed LFQ intensities, were considered statistically significant.

## Results

We detected a total of 210 proteins in all samples. There were 40 “unmapped” proteins that were not identified by Perseus. A full list of identified proteins is appended (Supplemental Table 1). We then examined the proteins of interest that were found to be significantly different in the pairwise comparisons of the groups.

### Change of protein expression as a function of age

Complement C1r was significantly decreased in serum from old rats compared with young (*p* < 0.05, Table 1). In addition, complement proteins complement 5 (C5), complement 6 (C6), and complement 8 gamma chain (C8) decreased with age (*p* < 0.05, Table 1). The protein ceruloplasmin, which is a copper transporter and antioxidant protein, was also found to be significantly decreased (*p* < 0.05, Table 1), which is consistent with the literature showing an age-dependent decrease in this protein and, interestingly, a decrease in this enzyme is associated with neurodegeneration (Musci et al. 1993; Connor et al. 1993; Jeong and David 2006). There was also a significant decrease in expression of insulin-like growth factor binding protein acid labile subunit (IGFALS) (*p* < 0.05, Table 1). It is interesting to note that with osteoporosis, there is a decrease in IGF-1 and its binding proteins in the serum (Fritton et al. 2010). Additionally, there was significantly decreased expression of maltase-glucoamylase (Mgam), which is consistent with age-dependent impaired digestion of carbohydrates (Fernandez-Alarcon et al. 2017).

**Table 1:** Expression of proteins in old rat serum compared with young

Symbol	Protein name	*p* value	Fold change
C1R	Complement C1r	0.0466	− 1.359
C5	Complement C5	0.0413	− 1.271
C6	Complement C6	0.00148	1.57
C8G	Complement C8 gamma chain	0.0287	− 1.399
CP	Ceruloplasmin	0.0358	− 1.299
CPN2	Caboxypeptidase N subunit 2	0.0168	− 1.473
GC	GC vitamin D binding protein	0.0164	1.201
IGFALS	Insulin like growth factor binding protein acid labile subunit	0.0341	− 1.305
Kng1/Kng2	Kininogen 2	0.0338	− 1.65
Mgam	Maltase-glucamylase	0.0236	− 1.799

**Fig. 1:**
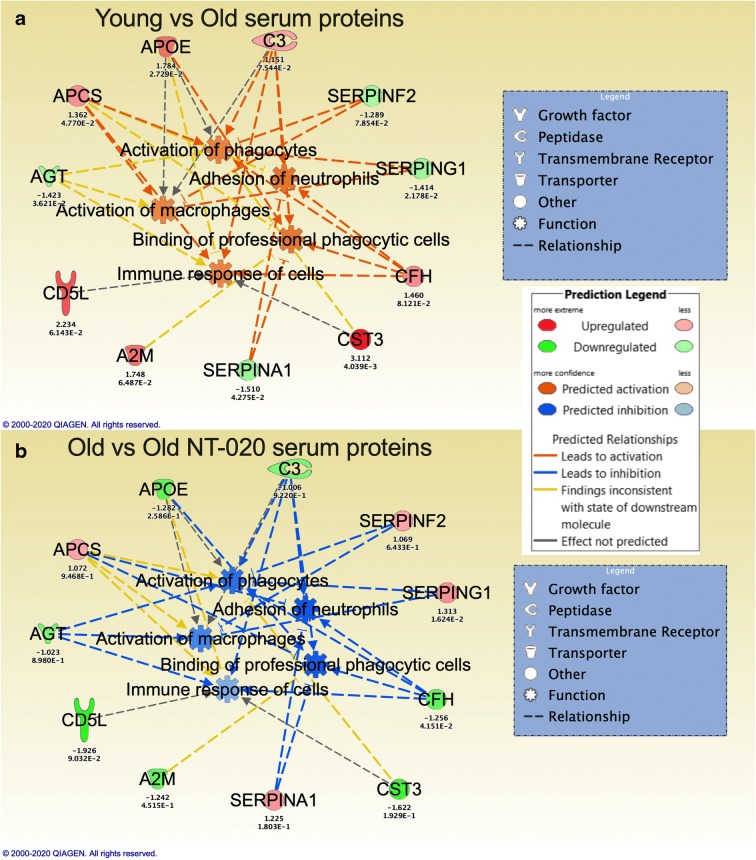
Predicted activation of cellular immune functions with age. As can be seen in this graphic representation of the predicted activation (orange) or inhibition (blue) of cellular immune functions, clustered in the middle of the graphic. In **a**, there is predicted upregulation of phagocytes, neutrophil adhesion, macrophages, binding of phagocytes, and immune response that is driven by the proteins detected in the old rat serum compared with young rat serum as shown in **a**. The proteins driving these predicted changes in cellular immune function are shown surrounding the functions. The fold change for protein expression is written below each protein along with the -log *p* value. As shown in the insert to the right, red indicates increased expression and green indicates decreased expression, and the intensity of the color reflects the degree of expression. For example, A2M is upregulated 1.75-fold in the aged rat serum, and CST3 is upregulated 3.11-fold. In **b**, the same proteins used in **a** were entered into IPA for the old vs old NT-020 treated comparison. As can be seen, the changes in protein expression following treatment with NT-020 now show a predicted inhibition of these same pathways in the old treatment group. For example, A2M is not − 1.24, and CST3 is − 1.62-fold. This shows an overall reduction in immune function predicted by the pattern of protein expression detected in the aged rat serum in the NT-020-treated animals. APOE, apolipoprotein E; C3, complement component 3; SERPINF2; SERPING1; CFH, complement factor H; CST3, cystatin 3; SERPINA1; A2M, alpha-2-macroglobulin; CD5L, CD5 molecule like; AGT, angiotensin; APCS, serum amyloid P component

In order to query how these changes affect functional pathways, the data was uploaded into Ingenuity Pathway Analysis (IPA, Qiagen) and the *p* value cutoff extended to *p* < 0.1 in order to increase the number of proteins for functional associations. The changes can be viewed graphically in Fig. 1a. This figure illustrates the changes with the highest *z* score all of which were related to immune function. Five cellular functions were top hits, and they are shown at the center of the figure. The proteins driving these findings are illustrated on the outside of the circle. The fold change is listed below each protein; green indicates a decrease and red an increase. As illustrated, all of the functional predictions are for an upregulation of immune responses including activation of macrophages and phagocytes.

### NT-020 counteracts upregulated immune response in aged rat serum

We next compared changes in the old animals following treatment with NT-020. All of the proteins that were entered into IPA for Fig. 1a were also uploaded for the ratios in the old diet treated rat serum versus old control diet to explore how diet altered the expression ratio. Only 7 of the 31 proteins that were significantly changed with age were significantly different with diet treatment at *p* < 0.05, and these are discussed below. For comparison with the change with age in immune function pathways, we illustrate the same function pathways in Fig. 1b with the same set of proteins used to generate Fig. 1a. As it is observed in Fig. 1b, all of the immune functional pathways that were upregulated with age are downregulated in the aged rats on the NT-020 diet. For example, cystatin 3 (CST3) changed from 3-fold increase with age to a − 1.6 decrease with diet treatment. CD5L was increased 2.2-fold with age and decreased 1.9-fold with NT-020 diet treatment. Overall, this led to a predicted functional downregulation of the immune response with NT-020 diet in the old rats.

### Autophagy pathway protein cathepsin A is upregulated with NT-020

The autophageal protein cathepsin A (CTSA) was shown to be significantly increased in serum from old rats given a diet supplemented with NT-020 (*p* < 0.05, Table 2). Autophagy is a turnover process for protein aggregates and damaged organelles. This process is considered protective and anti-aging, and inhibition or loss of autophagy occurs with age. Compromised autophagy has also been linked to neurodegenerative diseases and metabolic defects that are associated with aging (Martinez-Lopez et al. 2015; Boland et al. 2018). Autophagy is driven by a number of different proteins, among which are cathepsins. CTSA is a lysosomal protein that specifically triggers chaperone-mediated autophagy (CMA) through its serine carboxypeptidase activity, which triggers the degradation of lysosome-associated protein type 2a (Cuervo et al. 2003).

**Table 2:** Expression of proteins in old NT-020 serum versus old control

Symbol	Protein name	*p* value	Fold change
APOB	Apolipoprotein B	0.014	1.633
Apoc3	Apolipoprotein C-III	0.026	2.066
APOC4	Apolipoprotein C4	0.034	1.42
Apon	Apolipoprotein N	0.043	1.61
CFH	Complement factor H	0.042	− 1.256
CTSA	Cathepsin A	0.0085	2.965
LOC259246	Alpha-2u globulin PGCL1	0.0245	2.51
MST1	Macrophage stimulating 1	0.0319	− 1.361
SERPING1	Serpin family G member 1	0.0162	1.313

### Macrophage stimulating 1 decreases with NT-020

Macrophage stimulating 1/macrophage stimulating protein/hepatocyte growth factor-like protein (MST1/MSP/HGFL) expression was found to be significantly decreased with NT-020 treatment in old rats compared with old control (*p* < 0.05, Table 2). MST1 is a protein in serum that is predominantly secreted by the liver hepatocytes. Some studies have pointed to MSP as a negative regulator of inflammation and a key protein in maintaining metabolic homeostatis (Li et al. 2015). However, there is evidence that MST1/MSP activation increases pro-inflammatory cytokine production in the liver and lungs (Li et al. 2016; Wang et al. 2015). In the CNS, it has been found that there are receptors for MSP on microglia (Ron) and that MSP activation results in increased mRNA expression of pro-inflammatory cytokines (Suzuki et al. 2008).

### Complement system proteins were altered by NT-020

The complement system is part of the innate immune response and is considered protective for the retina with aging (Mukai et al. 2018). However, it has also been proposed that complement C1q promotes aging through interaction with the canonical Wnt signaling pathway (Naito et al. 2012). Additionally, early components of the compliment system have been shown to increase with age in a mouse model of AD (Reichwald et al. 2009). There are many proteins involved in the complement system, but two were found to be altered by NT-020: serpin G1, which increased with NT-020 treatment in old rats, and complement factor H (CFH), which decreased with NT-020 treatment in old rats (*p* < 0.05, Table 2). Both of these changes are consistent with an improved complement function. Serpin G1, also called C1 inhibitor or C1 esterase inhibitor, is largely considered to be a protective factor, and its inhibition has been associated with impaired blood-brain barrier (BBB) integrity and neuroinflammation in the brain (Farfara et al. 2019). Furthermore, deficient C1 inhibitor expression is linked to hereditary angioedema (Mete Gokmen et al. 2019), further suggesting a protective function.

Complement factor H (CFH) is an important regulator of the alternative pathway of the complement system. Interestingly, increased plasma CFH has been found to be correlated with geriatric depression (Shin et al. 2019). Additionally, genetic variants of CFH are strongly associated with age-related macular degeneration (AMD), and CFH has been shown to inhibit the anti-inflammatory activity of CD47 (Calippe et al. 2017). It has also been found that increased concentrations of complement 3 and CFH in the cerebrospinal fluid (CSF) of Parkinson’s disease, Alzheimer’s disease, and multiple-system atrophy patients correlated with disease severity (Wang et al. 2011).

## Discussion

Aging involves multiple complex physiological changes that lead to increased inflammation and loss of homeostasis and resilience and impaired regenerative capacity. The role of inflammation in aging has been widely researched and the term “inflammaging” has been coined to summarize how the inflammatory milieu leads to degeneration (Minciullo et al. 2016). While there is an increase in pro-inflammatory and other pro-aging factors and a decrease in anti-inflammatory factors with age, a complete profile of the aging milieu has yet to be identified, although parabiosis studies have contributed significantly to this effort (Villeda et al. 2011; Villeda et al. 2014; Smith et al. 2015). The current study sought to identify factors that were significantly changed with age and could be ameliorated by treatment with the polyphenol-rich dietary supplement NT-020, as NT-020 has been demonstrated to have beneficial effects in vitro and in vivo (Yasuhara et al. 2008; Shytle et al. 2010; Bachstetter et al. 2010; Kaneko et al. 2012; Small et al. 2014; Bickford et al. 2015; Flowers et al. 2016).Eleven proteins were found to change significantly when comparing old control rats with young controls. Of these, ceruloplasmin, insulin-like growth factor binding protein acid labile subunit (IGFALS), and maltase-glucoamylase (Mgam) were the most noteworthy. The protein ceruloplasmin, which is an enzyme that functions as a copper transporter and antioxidant protein, decreased significantly with age, which would be expected and, interestingly, a decrease in this enzyme is associated with neurodegenerative diseases (Musci et al. 1993; Connor et al. 1993; Jeong and David 2006). There was also a significant decrease in expression of IGFALS and decreased expression of IGF, and its binding proteins in serum have been linked with increased adipogenic potential of mesenchymal stem cells over osteogenic potential in osteoporosis (Fritton et al. 2010). Additionally, there was a significant decrease in expression of Mgam, which is consistent with age-dependent impaired digestion of carbohydrates and susceptibility to type 2 diabetes (Fernandez-Alarcon et al. 2017; Ren et al. 2011). Collectively, these data suggest that the significantly changed proteins were impacted by age as expected. However, the complement proteins were observed to decrease with age, whereas in the literature, they have been shown to increase with age (Gaya da Costa et al. 2018). While the reason for this disparity is unclear, the previously discussed proteins changed as expected with age. Further study is needed to determine whether this trend was global or tissue-specific, and a future study is ongoing, as later discussed.

Interestingly, when the data was entered into IPA and the *p* value cutoff extended to *p* < 0.1 in order to increase the number of proteins identified and query functional pathways, the results showed that the proteins identified drove upregulation of immune responses such as activation of macrophages and phagocytes with age and that all of these functional pathways became downregulated in the old NT-020 diet group. These data suggest that NT-020 was able to dampen the immune response in old rats.

Autophagy is the pathway by which protein aggregates and damaged organelles are degraded. With aging, there is decreased autophagy, leading to increased aggregation of proteins, which results in a toxic microenvironment and cellular dysfunction. There are three types of autophagy: macroautophagy, microautophagy, and chaperone-mediated autophagy (CMA). Altogether, autophagy is critical for protein and cellular homeostasis. The exact mechanisms underlying decreased autophagy with aging are unclear; however, it has been noted that autophagy is decreased in aging stem cell niches in particular, contributing to decreased regenerative capacity (Revuelta and Matheu 2017). There are many proteins that are involved in the autophagy pathway and among them are cathepsins. In the current study, cathepsin A (CTSA), which triggers CMA (Cuervo et al. 2003), was significantly increased in NT-020-treated old rats compared with age-matched controls.

Macrophage stimulating 1/macrophage stimulating protein/hepatocyte growth factor-like protein (MST1/MSP/HGFL) is a serum protein that is primarily secreted by hepatocytes in the liver. This protein has been speculated to have protective effects and to be a necessary regulator of metabolic activity (Li et al. 2015). However, recent studies have shown that MST1/MSP/HGFL increases pro-inflammatory cytokine production in the liver and lungs (Li et al. 2016; Wang et al. 2015). Non-alcoholic steatohepatitis (NASH) is a liver disease characterized by inflammation accumulation of lipids in the liver. NASH model mice treated with MSP were found to have significantly higher levels of tumor necrosis factor-α (TNF-α), chemokine (C-C motif) ligand 2 (Ccl2), intracellular adhesion molecule 1 (Icam1), interleukin 1beta (IL-1β), interferon gamma (IFNγ), B cell lymphoma 2 (Bcl2), macrophage markers F4/80, and cluster of differentiation 68 (CD68) (Li et al. 2016). Wang et al. exposed rats to combustion smoke in order to create a model of smoke-induced airway inflammation and found that MST1/MSP/HGFL and its receptor, RON, stimulated production of pro-inflammatory cytokines TNF-α, IL-8, IL-1β, and IL-10 (Wang et al. 2015). In the current study, MST1/MSP/HGFL decreased when old rats were given a diet supplemented with NT-020 compared with age-matched controls. Given the role of this protein in inflammation, this finding shows a mechanism by which NT-020 may resolve aspects of age-related inflammation.

The complement system constitutes part of the innate immune response. Some studies have shown that the complement system is protective against age-related retinal degeneration (Mukai et al. 2018). However, another study has shown that complement protein C1q promotes aging through activation of the canonical Wnt pathway (Naito et al. 2012). Furthermore, Reichwald et al. showed that expression of early complement proteins increased in an age-dependent manner and may contribute to AD pathogenesis in amyloid precursor protein-overexpressing mice (Reichwald et al. 2009). Together, these findings suggest that proteins from the complement system should be analyzed individually as to whether or not they are contributing to the aging phenotype. In the current study, we found changes in the complement proteins serpin G1/C1 inhibitor/C1 esterase inhibitor and CFH. Specifically, we observed a significant increase in the protective protein serpin G1 in old rats treated with NT-020. Knockdown of circulating plasma C1 inhibitor in the brain lead to decreased BBB-mediated extravasation and infiltration of plasma proteins and immune cells, activation of glial cells, impaired cognition, and depressive behavior in mice (Farfara et al. 2019). Interestingly, we also observed a significant decrease in CFH in serum from old rats given a diet supplemented with NT-020 compared with old rats given a standard diet. CFH is critical for activation of the alternate complement system. In a recent study, increased levels of CFH in plasma were positively correlated with depression in geriatric patients (Shin et al. 2019). Genetic variants of the CFH gene, which encodes the CFH protein, are associated with age-related macular degeneration (AMD). Calippe et al. (2017) also demonstrated that one AMD-associated CFH variant was able to suppress the anti-inflammatory activity of CD47. Another study examined the cerebrospinal fluid (CSF) samples taken from patients with Parkinson’s disease, Alzheimer’s disease, and multiple system atrophy and found that complement 3 (C3) and CFH were both increased, though to varying ratios depending upon the disease. Interestingly, both C3 and CFH concentrations correlated with disease severity in AD (Wang et al. 2011).

The results of the current study suggest that NT-020 is targeting multiple mechanisms associated with aging including autophagy and the complement system. However, the study was not without limitations. In spite of using a commercial kit to deplete the highly abundant albumin in our serum samples as well as the other most abundant proteins in serum, our detection of a low number of proteins suggests that albumin and other high-abundance proteins remained, preventing detection of other proteins in the samples. One reason for this could be that the depletion kit used was optimized for human tissue and not rat; however, there are no commercially available depletion kits with specificity for rat protein. Prior to opting to use this kit, we attempted to use an acid precipitation method but found it to be non-specific, leaving the possibility that we could be losing proteins we did not wish to deplete from the samples as well. Thus, we opted to use the human-optimized kit from Thermo Fisher Scientific, Inc. It is additionally worth noting that the proteins with a significant fold change that were detected in the old rats were not the same proteins found to have a significant fold change in the old rats given a diet supplemented with NT-020. Therefore, there was no detection of differential expression of individual proteins between these two groups. However, there were common pathways between them, most notably the complement system.

In addition, upon collection of serum, a protease inhibitor was not added prior to storing the samples at − 80 °C before use. This could also have led to protein degradation prior to analysis. Protease inhibitor was added after samples were thawed and processed for mass spectrometry; however, this step may have been undertaken later than would have been optimal.

In spite of some of the methodological challenges involved with working with serum, these findings contribute to the existing body of knowledge on how nutraceuticals impact the aging milieu and exert therapeutic effects. In particular, we have found that NT-020 reverses the negative effects of age on stem cell proliferation, enhances neurogenesis, and leads to improved performance on cognitive behavioral paradigms (Acosta et al. 2010; Bachstetter et al. 2010; Bickford et al. 2015; Flowers et al. 2015; Flowers et al. 2016; Kaneko et al. 2012; Shytle et al. 2010; Small et al. 2014; Yasuhara et al. 2008). These data provide insight into the mechanisms of action underpinning NT-020-mediated therapeutic effects. Additionally, future studies are needed to compare the proteome from serum to that of CNS tissue or cells in order to determine whether circulating factors in the periphery have an impact on the brain. A study is currently in progress to compare the results of the serum analysis with microglia from the same animals.

## Electronic supplementary material

ESM 1 (XLSX 33 kb)
